# Progressive Multifocal Leukoencephalopathy with Negative JC Virus PCR following Treatment of Follicular Lymphoma: Implications for Biologics in the Era of Targeted Cancer Therapy

**DOI:** 10.1155/2015/534529

**Published:** 2015-12-15

**Authors:** Kimberly A. Silverio, Shyam A. Patel

**Affiliations:** ^1^Department of Gynecologic Oncology, Helen Diller Family Comprehensive Cancer Center, University of California, San Francisco (UCSF), 550 16th Street, San Francisco, CA 94158, USA; ^2^Department of Medicine, Stanford University Medical Center, 300 Pasteur Drive, Stanford, CA 94305, USA

## Abstract

Anticancer therapy predisposes patients to infections by the immunosuppression that results from treatment. Although 85% of patients with progressive multifocal leukoencephalopathy (PML) have concurrent HIV/AIDS, PML can also develop in patients after they receive chemotherapy for cancer. The case herein describes a 69-year-old man with history of follicular lymphoma who presented with progressive dysarthria and right-sided paralysis. He received rituximab one year prior to presentation. PET scan suggested no recurrence of lymphoma. Cerebrospinal fluid (CSF) analysis was negative and showed fewer than 500 copies/mL of JC virus. However, brain biopsy showed chromatin margination and viropathic change within oligodendrocytes, confirming PML. He was started on mirtazapine and mefloquine with some clinical improvement. To our knowledge, this is the first case of rituximab-associated PML in a patient with negative JC virus PCR from the CSF. Recognition of PML in the differential of oncology patients with CNS symptoms is an important consideration as we enter the era of targeted therapy and personalized cancer medicine involving biologics. Furthermore, screening of patients for presence of subclinical JC viremia prior to the use of biologics may be an important component of assessing patient candidacy for these agents.

## 1. Introduction

Novel treatment modalities for cancer, including biologic agents such as monoclonal antibodies, have resulted in remarkable improvements in clinical outcomes for patients with cancer over the recent two decades. Targeted therapy and personalized cancer medicine have led to increased treatment efficacy with reduced toxicity. However, novel modalities of treatment do not remain without complications. As targeted therapy for cancer has only been on the market since approximately the 1990s, we do not have enough robust data on adverse effects and management of these complications. Some recent literature has underscored the potentially adverse effects of rituximab in treatment of non-Hodgkin's lymphoma, namely, PML. Complications such as these have significant implications as we approach an era of personalized cancer medicine, as the toxicity of novel agents typically presents with more subtle and elusive symptomatology compared to conventional chemotherapy. Such symptomatology is rooted in the specific mechanism of action of targeted agents. The clinical significance, however, is of utmost importance. This case describes a patient with a subacute presentation of a complication characterized by high mortality in the setting of treatment for follicular lymphoma with a biologic agent.

## 2. Case Description

**Figure 1:**
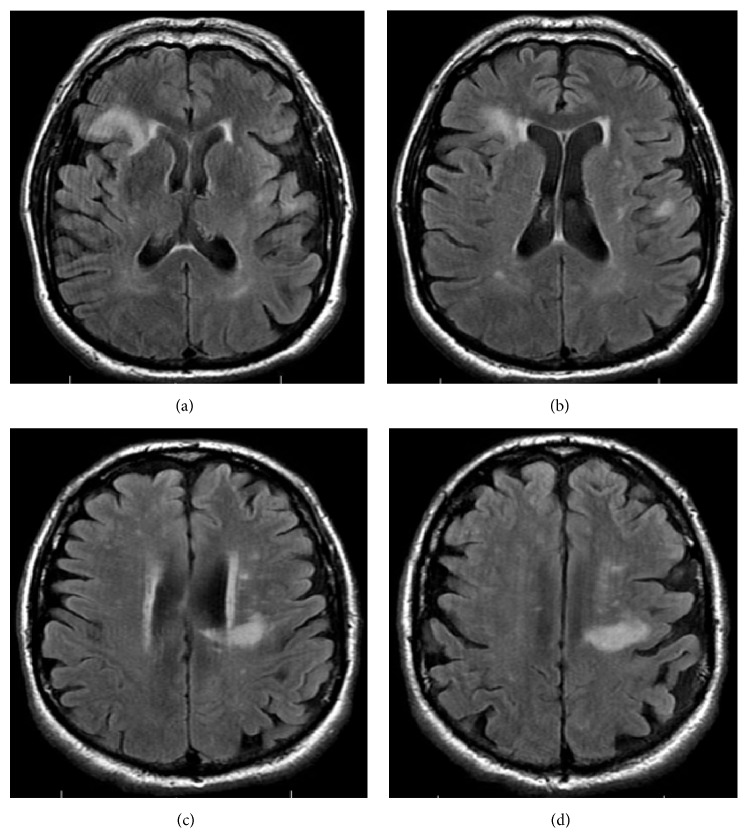
Axial images of MRI of the brain showing multiple confluent foci of T2-weighted FLAIR hyperintensity involving the inferior right and left frontal lobes, as well as periventricular regions (a-b). Hyperintensities are also seen in the left corona radiata (c-d). These areas are representative CNS demyelination. Several foci had restricted diffusion. There was no flow-limiting stenosis, occlusion, or aneurysm in intracranial and extracranial circulations. Images progress superiorly to inferiorly from (a) through (d).

**Figure 2:**
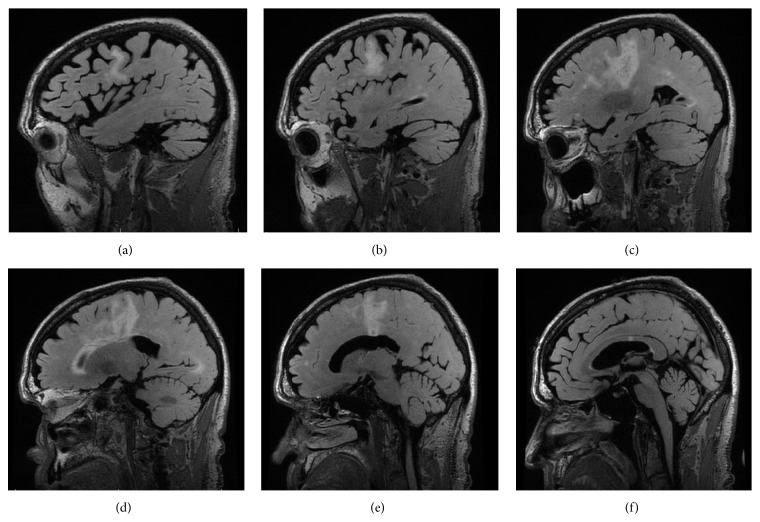
Sagittal images of MRI of the brain with T2-weighted FLAIR hyperintensities. Images progress from left to right from (a) through (f). Areas of demyelination correspond anatomically with axial images from Figure 1.

**Figure 3:**
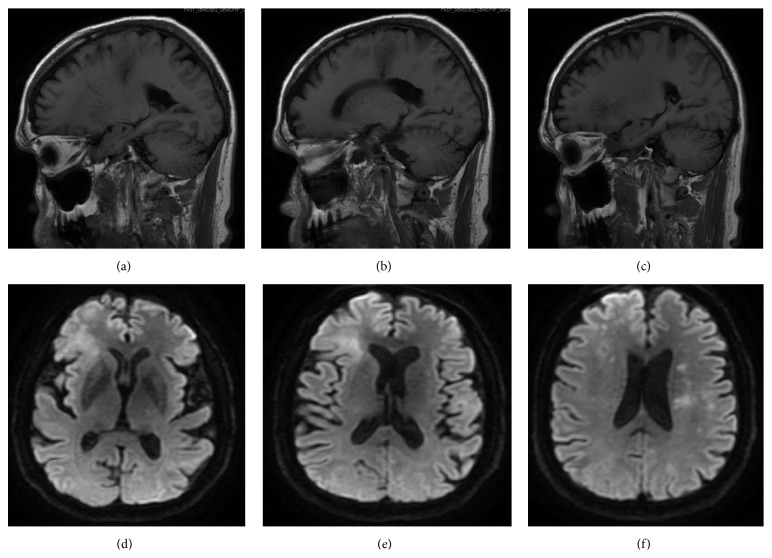
T1-weighted sagittal images (a–c) and diffusion-weighted images (DWI) (d–f). Images progress from right to left from (a) through (c). Images progress superiorly to inferiorly from (d) through (f).

**Figure 4:**
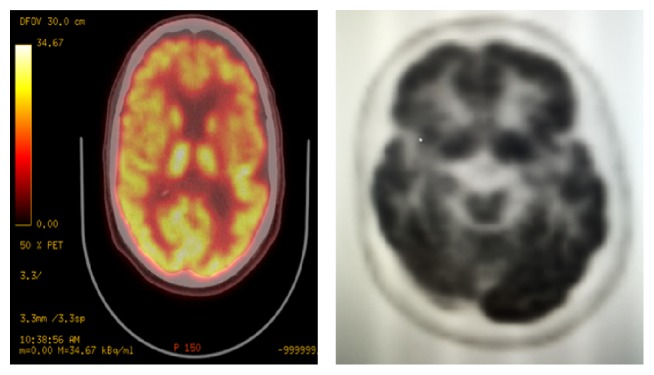
PET scan of the brain showing no hypermetabolic lesions in the brain to suggest lymphoma. Hypometabolism is seen in the right frontal operculum and left frontal vertex. There is a larger area of subtle asymmetric decreased FDG activity seen in posterior left frontal lobe and left parietal lobe.

**Figure 5:**
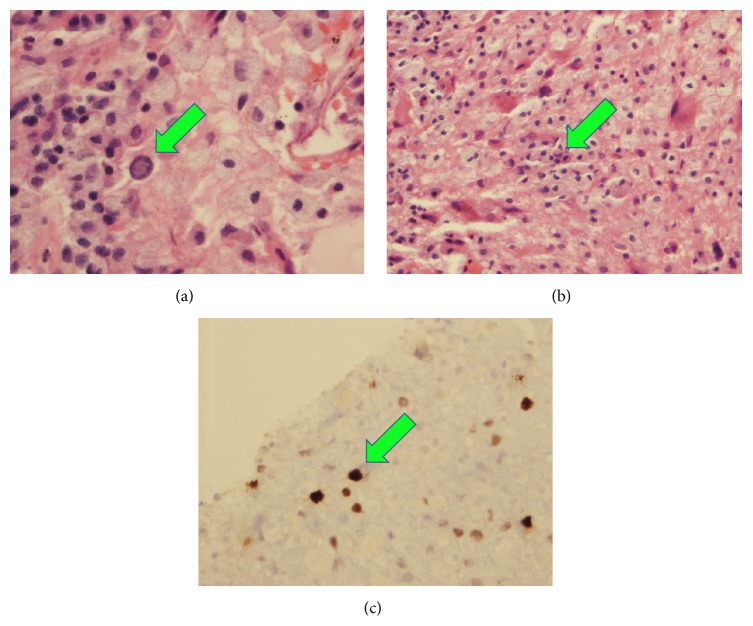
Frontal lobe biopsy of the brain showing chromatic margination in oligodendrocytes (a), perivascular mixed chronic inflammation (b), and SV40 antigen positivity (c) consistent with JC virus infection. The aforementioned histological findings are demarcated by green arrows.

A 69-year-old man with a history of stage IIIB follicular lymphoma, atrial fibrillation, and pulmonary sarcoidosis presented with progressive dysarthria and right-sided paralysis over a two-month period. The patient noticed increasing difficulty with speech and fine-motor function such as handwriting and finally decided to seek evaluation due to nonresolving symptoms. Regarding relevant past medical history, he was diagnosed with follicular lymphoma 20 years prior to presentation and had three recurrences in the interim. He completed six cycles of rituximab and bendamustine one year prior to presentation for his lymphoma. Upon admission to the hospital for progressive neurological symptoms, workup was initiated. There was high concern for cerebrovascular etiology versus recurrence of lymphoma with CNS involvement. MRI of the brain showed multiple confluent foci of T2-weighted FLAIR hyperintensity involving the inferior frontal lobes and left corona radiata, initially suggestive of an inflammatory, neoplastic, or vasculitic process. These hyperintensities were noted on axial images (Figure 1) and sagittal images (Figure 2). There was no flow-limiting stenosis, occlusion, or aneurysm. Corresponding T1-weighted sagittal images and DWI sequences are shown for comparison (Figure 3). His lab values (including CBC and metabolic panel) on admission were unremarkable. Rheumatologic workup was done: ANA was negative, ANCA titer was < 1 : 20, complement C3 was 104 mg/dL (within normal limits), and complement C4 was 23 mg/dL (within normal limits). Workup for inflammatory etiology showed CRP of 0.16 mg/dL and ESR of 17 mm/hr. Workup for neurosarcoidosis showed ACE level of 27 mcg/L (within normal limits). Workup for immunodeficiency showed nonreactive HIV screen, nonreactive HCV screen, IgA level of 270 mg/dL, IgG level of 836 mg/dL, and IgM level of 44 mg/dL (all within normal limits). Syphilis ELISA was < 0.8, and Lyme antibody screen was < 0.9. Workup for possible recurrence of lymphoma showed beta2-microglobulin of 2.28 mcg/mL and LDH of 109 U/L (within normal limits). PET scan revealed no hypermetabolic lesions in the brain to suggest recurrence of his lymphoma (Figure 4). Skin biopsy was done and was negative for intravascular lymphoma. Finally, lumbar puncture was pursued, and CSF was clear and colorless. Other studies of the CSF showed WBC, 0; RBC, 0; glucose, 57 mg/dL; protein, 38 mg/dL; no organisms; and VDRL, nonreactive. Blood cultures remained negative. CSF PCR was negative for JC virus PCR (<500 copies/mL). Due to concern for inflammatory etiology of his symptoms, he was started empirically on high-dose IV methylprednisolone, but there was no subsequent improvement. A repeat MRI of the brain showed mildly increased size of several nonenhancing confluent subcortical and periventricular T2/FLAIR signal hyperintensities mostly in left corona radiata and right temporal lobe with associated foci of restricted diffusion. There were scattered punctate foci of enhancement in the bilateral cerebral hemispheres not definitively seen on the prior MRI. Ultimately, a frontal lobe brain biopsy was performed given the elusive diagnosis. The brain biopsy revealed perivascular mixed chronic inflammation, chromatin margination with viropathic change within oligodendrocytes, demyelination, and SV40 stain positivity confirming JC virus infection (Figure 5). The patient was started in mefloquine and mirtazapine, given the recent data on this regimen in non-HIV/AIDS-related PML. He had clinical improvement and is currently being managed as an outpatient.

## 3. Discussion

PML is a demyelinating disease that affects oligodendrocytes and can lead to significant morbidity and mortality. The infectious agent is JC virus, which is a papovavirus first isolated by John Cunningham in 1971. JC virus is a lytic virus in oligodendrocytes [1]. PML was previously thought to occur exclusively in patients with HIV/AIDS (CD4 < 500/mcl), and approximately 85% of cases have coexisting HIV infection. PML is in fact an AIDS-defining illness. However, PML development is not exclusive to HIV/AIDS patients. Other risk factors include hematological and lymphoreticular malignancies (resulting in inherent immunosuppression) and receipt of agents such as cyclophosphamide, prednisone, and rituximab (resulting in medication-induced immunosuppression).

The pathophysiology of PML development in patients with cancer who have received rituximab involves reactivation of endemic JC virus [2]. The virus enters the body via the oral or respiratory route. It establishes latency in the kidneys and lymphoreticular tissues. Importantly, the virus can replicate without causing clinical manifestations, as was the case here. Immunosuppression causes reactivation and dissemination to the brain by B cells, which serve as reservoirs of the virus. Infected astrocytes acquire a bizarre shape and distortion of nuclei, and demyelination results in diverse neurological symptoms.

Monoclonal antibody therapy has been linked to PML in the past decade. The integrin inhibitor natalizumab, for example, has been associated with PML via a mechanism that likely involves disruption of inflammatory T cell extravasation [3]. The link between PML and rituximab is less well characterized but has been described in some case reports. One of the first reports on PML development after rituximab treatment was in 2007 [4]. This was a case in which a 44-year-old female was diagnosed with PML two years after receiving rituximab and then died. PCR of JC virus from the CSF was positive. In another case, a 48-year-old female developed PML after 5 cycles of R-CHOP chemotherapy, and JC virus testing by PCR of CSF was positive [5]. In a report of 57 cases of postrituximab PML, JC viral PCR of CSF was used as a confirmatory criterion for PML [6].

A striking and clinically significant aspect of the case described here was that the patient's JC virus PCR from CSF was negative. JC virus PCR from the CSF has a sensitivity of 74–93% and specificity of 92–99% for PML [7]. Potential etiologies for false negative PCR include low viral load in CSF, storage of specimen prior to testing, low volume specimen, or loss of DNA during concentration. The case herein highlights the concept that a diagnosis of PML can be made in the absence of CSF PCR positivity. Specifically, radiographic (i.e., MRI) and pathologic (i.e., brain biopsy) findings are sufficient for diagnosis. This is especially important because prior case studies have used JC virus CSF PCR as a confirmatory criterion for PML [6].

From a practical perspective, it is important to identify which subsets of patients may be at risk for PML after receiving biologics. JC viremia is common in the general population, but PML does not develop in healthy persons due to absence of immunosuppression. However, in patients who become immunosuppressed, the presence of JC viremia is a risk factor for PML because viral reactivation can occur. This underscores the importance of a secondary prevention strategy, namely, testing for JC viremia prior to starting biologics. For example, in patients who are candidates for natalizumab for multiple sclerosis, the potential for JC virus reactivation can be assessed by testing for JC virus exposure via antibody index or real-time PCR of plasma or urine [8]. In the same respect, for patients who are candidates for biologics for cancer, PCR of JC virus from the plasma may be a useful screening measure prior to initiation of immunosuppressive biologics, and these can divert adverse events.

Recent advances in the treatment options for non-HIV/AID-associated PML have been made in the past six years. The use of mefloquine and mirtazapine for non-HIV/AIDS-related PML came to the forefront after screening FDA-approved agents for activity against JC virus. Screening of approximately 2000 drugs showed that mefloquine, a known antimalarial agent, harbored the ability to inhibit JC virus infection by inhibiting viral replication after cell entry [9]. The use of mirtazapine, which has serotonin receptor inhibitory activity, came to attention when it was discovered that JC virus uses the serotonin receptor to gain cell entry [10]. The patient in this case showed improvement after starting this regimen.

In summary, this case report highlights the need to maintain a high index of suspicion for opportunistic infections in cancer patients without HIV/AIDS. Conventional chemotherapy, namely, antimetabolites and DNA-disrupting agents, has long been recognized to cause immunosuppression with resulting complications. However, novel anticancer agents have not been available for enough time to appreciate the potential complications. Novel agents that may have the potential to predispose patients to subacute infections or inflammatory complications include monoclonal antibody therapy that depletes immune cells, immune checkpoint inhibitors, and antigrowth factor therapy. This case emphasizes the need to recognize PML in the differential diagnosis of cancer patients with neurological symptoms, regardless of whether they had received conventional chemotherapy or novel agents. The case underscores the diagnostic limitations such as sensitivity of CSF viral PCR testing for JC virus; leptomeningeal detection JC virus is not a prerequisite for PML. Negative JC viral PCR testing should be followed by ultrasensitive PCR or by brain biopsy. Empiric treatment for PML should be considered given the minimal risks of regimens that have been proven effective, such as mirtazapine and mefloquine, and given that PML can be rapidly fatal once diagnosed [11, 12]. Finally, this case highlights the utility of screening for JC viremia in patients who may be candidates for novel biologics for cancer treatment.
